# New Bergamotane Sesquiterpenoids from the Plant Endophytic Fungus *Paraconiothyrium brasiliense*

**DOI:** 10.3390/molecules200814611

**Published:** 2015-08-12

**Authors:** Zhe Guo, Fengxia Ren, Yongsheng Che, Gang Liu, Ling Liu

**Affiliations:** 1 State Key Laboratory of Mycology, Institute of Microbiology, Chinese Academy of Sciences, Beijing 100190, China; E-Mail: guozhe12@mails.ucas.ac.cn; 2 State Key Laboratory of Toxicology & Medical Countermeasures, Beijing Institute of Pharmacology & Toxicology, Beijing 100850, China; E-Mails: ren2006victory@126.com (F.R.); cheys@im.ac.cn (Y.C.); 3 University of Chinese Academy of Sciences, Beijing 100049, China

**Keywords:** *Paraconiothynium brasiliense*, endophytic fungus, sesquiterpenoid

## Abstract

Brasilamides K-N (**1**–**4**), four new bergamotane sesquiterpenoids; with 4-oxatricyclo (3.3.1.0 ^2,7^)nonane (**1**)and 9-oxatricyclo(4.3.0.0 ^4,7^)nonane (**2**–**4**) skeletons; were isolated from the scale-up fermentation cultures of the plant endophytic fungus *Paraconiothynium brasiliense* Verkley. The previously identified sesquiterpenoids brasilamides A and C (**5** and **6**) were also reisolated in the current work. The structures of **1**–**4** were elucidated primarily by interpretation of NMR spectroscopic data. The absolute configurations of **1**–**3** were deduced by analogy to the co-isolated metabolites **5** and **6**; whereas that of C-12 in **4** was assigned using the modified Mosher method. The cytotoxicity of all compounds against a panel of eight human tumor cell lines were assayed.

## 1. Introduction

**Figure 1:**
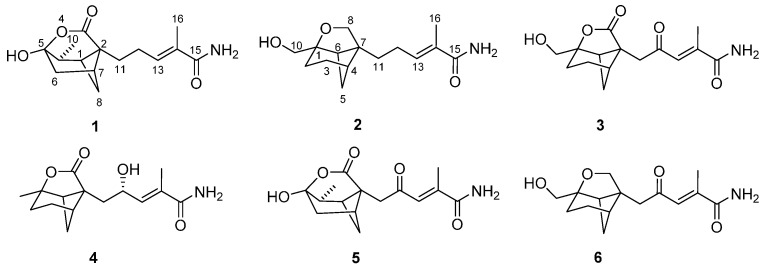
Structures of compounds **1**–**6**.

Sesquiterpenoids incorporating the bergamotane skeleton have been reported from various natural sources. Fungus-derived bergamotane metabolites include pinthunamide, tricyclic sesquiterpene amide isolated from *Ampulliferina* sp. [1]; ampullicin, isoampullicin, and dihydroampullicin, plant growth regulators from *Ampulliferina*-like sp. No. 27 [2,3]; the expansolides, tetracyclic sesquiterpene lactones from *Penicillumexpansum* and *Aspergillus fumigatus* Fresenius [4], and the massarinolins, antibacterial agents from the aquatic fungus *Massarina tunicate* [5]. Endophytic fungi, which inhabit normal tissues of hosts without causing apparent symptoms of pathogenesis, are rich sources of bioactive natural products [6,7,8,9,10,11,12]. In an ongoing search for new bioactive secondary metabolites, a strain of *Paraconiothyrium brasiliense* Verkley (M3-3341) isolated from the branches of* Acer truncatum* Bunge on Dongling Mountain, Beijing, China, was investigated, leading to the discovery of brasilamides A–D, four new bergamotane sesquiterpenes with anti-HIV-1 activity [13]. Subsequent chemical investigations of the extract from a larger-scale fermentation of this fungus led to the isolation of cytotoxic bisabolane sesquiterpeniods [14]. Further separation of the remaining fractions afforded brasilamides K-N (**1**–**4**), four new bergamotane sesquiterpenoids with 4-oxatricyclo(3.3.1.0 ^2,7^)nonane (**1**) and 9-oxatricyclo (4.3.0.0 ^4,7^)nonane (**2**–**4**) skeletons (Figure 1), respectively, together with brasilamides A and C (**5** and **6**) [13]. Details of the isolation, structure elucidation, and cytotoxicity of these metabolites are reported herein.

## 2. Results and Discussion

**Figure 2:**
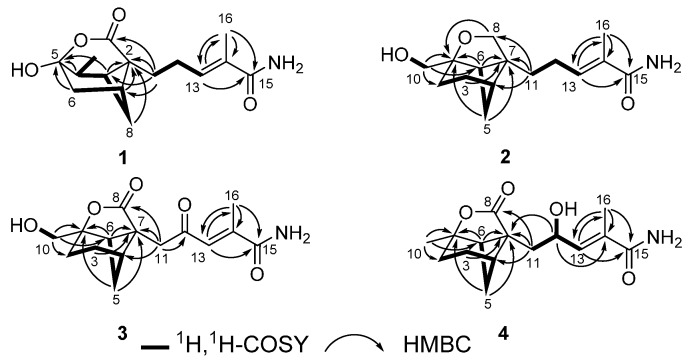
The ^1^H-^1^H-COSY and selected HMBC (H→C) correlations for **1**–**4**.

**Figure 3:**
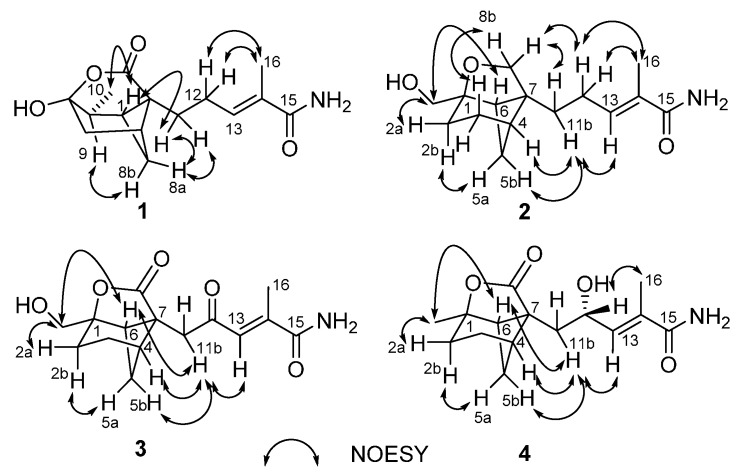
NOESY correlations for **1**–**4**.

Brasilamide K (**1**) gave a pseudomolecular ion [M + H]^+^ peak by HRESIMS, corresponding to a molecular formula of C_15_H_21_NO_4 _(six degrees of unsaturation). Analysis of its ^1^H- and ^13^C-NMR data (Table 1) revealed the presence of three exchangeable protons (δ_H_ 6.74, 6.38, and 6.07, respectively), two methyl groups, four methylene units, three methines, two sp^3^ quaternary carbons (one oxygenated), one trisubstituted olefin, and two carboxylic carbons (δ_C_ 172.9 and 170.9, respectively). The ^1^H-^1^H COSY NMR data showed two isolated spin-systems of C-6–C-10 (via C-7, C-8, C-1, and C-9) and C-11–C-13. HMBC correlations (Figure 2) from H-13 to C-15 and C-16, and from H_3_-16 to C-13, C-14, and C-15 enabled both methyl carbon C-16 and carboxylic carbon C-15 attached to the C-14 of C-13/C-14 olefin. HMBC cross-peaks from H-1, H_2_-6, and H-9 to C-2 and C-5, plus H_2_-8 to C-2 indicated that the sp^3^ quaternary carbon C-2 is located between C-1 and C-7, whereas the C-5 oxygenated sp^3^ quaternary carbon is attached to both C-6 and C-9, completing the bicyclo(3.1.1)heptane ring, while those from H_2_-11 to C-1, C-2, C-3, and C-7 led to the connection of C-2 to C-3 and C-11. Considering the doubly-oxygenated nature of C-5 (δ_C_ 103.0) and the unsaturation requirement of **1**, the C-3 carboxylic carbon must acylate one of the oxygen atoms attached to C-5 to form a δ-lactone moiety, thereby completing the 4-oxatricyclo(3.3.1.0 ^2,7^)nonane skeleton in **1**. The remaining two exchangeable protons were assigned as 15-NH_2_, by default. Collectively, these data permitted assignment of the planar structure of **1**. The relative configuration of **1** was deduced to be the same as brasilamide A (**5**) by comparison of its NOESY data (Figure 3) with those of **5** [13]. NOESY correlations of H-1 withH_3_-10 and H_2_-11, H-8a with H_2_-11, and H-8b with H-9 revealed their proximity in space. The C-13/C-14 olefin is assigned the *E*-geometry based on NOESY correlations of H_2_-12 with H_3_-16. The absolute configuration of **1** was proposed as 1*S*,2*S*,5*R*,7*R*,9*S* by analogy to those of **5**, which was secured by X-ray crystallography [13].

**Table 1:** NMR Data for **1** and **2** (Acetone-*d*_6_).

Pos.	1			2		
	δ_C_^a^	δ_H_^b^ (*J* in Hz)	HMBC	δ_C_^a^	δ_H_^b^ (*J* in Hz)	HMBC
1	43.3	2.00, t (6.0)	2, 5, 7, 8, 10, 11	89.8		
2	47.0			28.8	1.48, m; 1.90, m	1, 3, 4, 6, 10
3	172.9			23.3	1.76, m; 1.83, m	1, 2, 4, 5, 7
4				40.5	2.22, m	2, 6, 11
5	103.0			23.8	1.47, d (11.0); 2.10, ddd (11.0, 6.0, 5.5)	1, 3, 4, 6, 7, 8
6	41.9	2.09, m	2, 5, 8	48.0	2.27, dd (5.5, 5.0)	1, 2, 4, 7, 10, 11
7	36.2	2.36, m	1, 8	56.0		
8	37.1	1.31, d (11.0); 2.70, ddd (11.0, 7.0, 6.0)	1, 2, 3, 6, 7, 9	71.5	3.46, d (9.0); 3.80, d (9.0)	1, 4, 6, 7, 11
9	44.4	2.24, q (7.0)	1, 2, 5, 8, 10			
10	12.9	0.89, d (7.0)	1, 5, 9	67.2	3.40, d (12.0); 3.43, d (12.0)	1, 2, 6
11	30.1	1.95, m	1, 2, 3, 7, 12, 13	33.6	1.68, m; 1.85, m	4, 6, 7, 8, 12, 13
12	25.5	2.34, m	2, 11, 13, 14	25.2	2.04, m; 2.16, m	11, 13, 14
13	136.0	6.45, t (7.5)	12, 15, 16	136.1	6.40, t (7.5)	11, 12, 15, 16
14	132.0			131.8		
15	170.9			170.9		
16	12.7	1.82, s	13, 14, 15	12.6	1.80, s	13, 14, 15
OH-5		6.38, s				
OH-10					3.32, brs	
NH_2_-15		6.74, brs; 6.07, brs			6.73, brs; 6.09, brs	

^a^ Recorded at 150 MHz; ^b^ Recorded at 600 MHz.

Brasilamide L (**2**) was assigned the molecular formula C_15_H_23_NO_3_ (five degrees of unsaturation) by HRESIMS (266.1743). Interpretation of its ^1^H- and ^13^C-NMR data (Table 1) revealed the presence of three exchangeable protons (δ_H_ 6.73, 6.09, and 3.32, respectively), one methyl group, seven methylene units (two oxygenated), two methines, two sp^3^ quaternary carbons (one oxygenated), one trisubstituted olefin, and one carboxylic carbons (δ_C_ 170.9). The ^1^H-^1^H COSY NMR data showed three isolated spin-systems of C-2–C-6, C-10–OH, and C-11–C-13. HMBC cross-peaks (Figure 2) from H-13 to C-15 and C-16, and from H_3_-16 to C-13, C-14, and C-15 enabled both methyl carbon C-16 and carboxylic carbon C-15 attached to the C-14 of C-13/C-14 olefin. HMBC correlations from H_2_-3, H_2_-5, and H-6 to C-1 and C-7, plus H_2_-10 to C-1, C-2, and C-6 indicated that the sp^3^ quaternary carbon C-7 is located between C-4 and C-6, and C-2, C-6, and C-10 are all connected to the C-1 oxygenated sp^3^ quaternary carbon, completing the bicyclo(3.1.1)heptane ring. While those from H_2_-11 to C-4, C-6, C-7, and C-8 led to the connection of C-7 to C-8 and C-11. A key correlation from H_2_-8 to C-1 established the furan ring, thereby completing the 9-oxatricyclo(4.3.0.0 ^4,7^)nonane skeleton in **2**. The remaining two exchangeable protons were also assigned as 15-NH_2_ by default. On the basis of these data, the gross structure of **2** was established as shown. NOESY correlations (Figure 3) of H-3b with H-8b, H-4 and H-5b with H-11b, and H-6 with H_2_-10 established the relative configuration of **1**, which is the same as that of brasilamide C (**6**) by comparison of their NOESY data with those of **6** [13]. The C-13/C-14 olefin is also assigned the *E*-geometry based on NOESY correlations of H_2_-12 with H_3_-16. The absolute configuration of **2** could be deduced as shown by analogy to **6**.

The elemental composition of brasilamideM (**3**) was established as C_15_H_19_NO_5_ (seven degrees of unsaturation) by HRESIMS (*m*/*z* 294.1332 [M + H]^+^), 28 mass units higher than **2**. The ^1^H-, and ^13^C-NMR spectra (Table 2) of **3** displayed signals for structural features similar to **2**, except that two methylene units (δ_H/C_ 3.46, 3.80/71.5; 2.04, 2.16/25.2) in **2** are replaced by one carboxy carbon atom (δ_C_ 177.8) and one α,β-unsaturated ketone carbon atom (δ_C_ 199.5) in the spectra of **3**, respectively. It was confirmed by HMBC correlations (Figure 2) from H_2_-11 to C-8 and C-12. Analysis of NOESY data (Figure 3) of **3** revealed the same relative configuration as **2**, implying that its absolute configuration could be deduced as shown by analogy to **2**.

**Figure 4:**
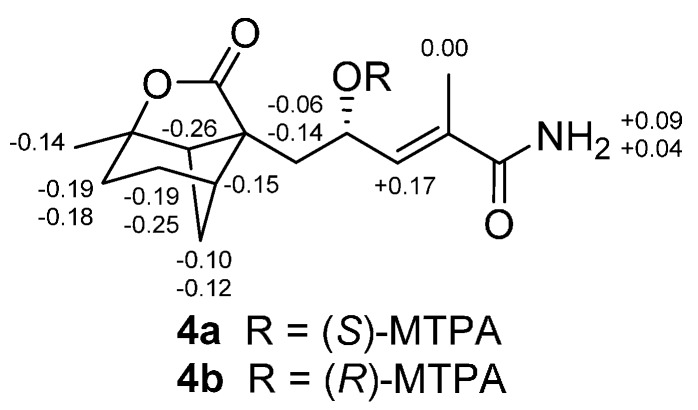
∆δ Values (in ppm) = δ*_S_* − δ*_R_* obtained for (*S*)- and (*R*)-MTPA esters **4b** and **4a**.

The molecular formula of brasilamideN (**4**) was determined to be C_15_H_21_NO_4_ (six degrees of unsaturation) on the basis of its HRESIMS (*m*/*z* 280.1546 [M + H]^+^). Interpretation of its NMR data (Table 2) revealed structural features similar to those presented in **3**, except that the oxygenated methylene unit (δ_H/C_ 3.70/66.4) and the C-10 carboxy carbon (δ_C_ 199.5) are replaced by the methyl (δ_H/C_ 1.42/25.1) and the oxygenated methine unit (δ_H/C_ 4.57/67.1), respectively. This observations were confirmed by ^1^H-^1^H COSY and HMBC correlations (Figure 2) from H_3_-10 to C-1, C-2 and C-6, H-12 to C-7, C-11, C-13, and C-14. The relative configuration of **4** was deduced to be the same as those of **3** on the basis of its NOESY data (Figure 3) except the stereogenic center C-12. The absolute configuration of C-12 in **4** was assigned by application of the modified Mosher method [15]. Treatment of **4** with (*S*)-MTPA Cl and (*R*)-MTPA Cl afforded the *R*-MTPA ester (**4a**) and *S*-MTPA ester (**4b**), respectively. The difference in chemical shift values (∆δ = δ*_S_* − δ*_R_*) for the diastereomeric *S*-MTPA (**4b**) and *R*-MTPA (**4a**) esters (Figure 4) was calculated to assign the 12*S* absolute configuration. The absolute configuration of **4** was deduced as shown by analogy to **3**.

**Table 2:** NMR Data for **3** and **4** (Acetone-*d*_6_).

Pos.	3			4		
	δ_C_^a^	δ_H_^b^ (*J* in Hz)	HMBC ^a^	δ_C_^c^	δ_H_^d^ (*J* in Hz)	HMBC ^c^
1	90.7			88.7		
2	26.0	1.84, m; 2.12, m	1, 3, 4, 6	30.5	1.78, m; 2.06, m	1, 3, 4, 6
3	23.2	1.77, m; 1.96, m	1, 2, 4, 5, 7	23.8	1.77, m; 1.94, m	1, 2, 4, 5, 7
4	41.6	2.43, m	2, 3, 5, 6, 7, 11	42.7	2.38, m	2, 6, 7, 11
5	23.5	1.72, d (11.0); 2.30, ddd (11.0, 6.0, 5.5)	1, 3, 4, 6, 7, 8	23.2	1.76, d (11.0); 2.35, ddd (11.0, 6.0, 5.5)	1, 3, 4, 6, 7, 8
6	44.3	2.82, dd (5.5, 5.0)	1, 2, 4, 7, 10, 11	48.7	2.90, dd (6.7, 5.5)	1, 2, 4, 5, 10, 11
7	53.6			55.9		
8	177.8			179.1		
10	66.4	3.70, brs	1, 2, 6	25.1	1.42, s	1, 2, 6
11	44.5	3.09, d (19.0); 3.15, d (19.0)	4, 6, 7, 8, 12	37.3	1.89, m; 1.99, m	4, 6, 7, 8, 12, 13
12	199.5			67.1	4.57, ddd (10.7, 10.6, 5.2)	7, 11, 13, 14
13	128.2	6.82, q (1.5)	12, 14, 15, 16	139.1	6.25, dq (10.6, 1.5)	11, 14, 15, 16
14	146.2			131.3		
15	170.6			171.3		
16	14.9	2.15, d (1.5)	13, 14, 15	13.2	1.83, d (1.5)	13, 14, 15
OH-10		4.10, brs				
OH-12					4.15, brs	11
NH_2_-15		7.18, brs; 6.62, brs			6.73, brs; 6.19, brs	

^a^ Recorded at 100 MHz; ^b^ Recorded at 400 MHz; ^c^ Recorded at 125 MHz; ^d^ Recorded at 500 MHz.

Compounds **1**–**6** were tested for cytotoxicity against a panel of eight human tumor cell lines A549 (human lung adenocarcinoma cells), A375 (human malignant melanoma cells), MCF-7 (human breast cancer cells), CNE1-LMP1 (stable oncoprotein LMP1 integrated nasopharyngeal carcinoma cells), EC109 (human esophageal cancer cells), MGC (human gastric cancer cells), PANC-1 (human pancreatic carcinoma cells), and Hep3B-2 (human hepatoma carcinoma cells). Unfortunately, compounds **1**–**6** did not show detectable cytotoxicity at 50 µM.

Bergamotane sesquiterpenoids incorporating 4-oxatricyclo(3.3.1.0 ^2,7^)nonane skeletonare rare. Precedents include brasilamides A and B, which were isolated from *P. brasiliense* in our previous study [13]. Structurally, brasilamide K (**1**) is a hydrogenated analogue of brasilamide A (**5**) [13], with a tetrahydro-2*H*-pyrone moiety attached to the bicyclo(3.1.1)heptane ring at C-2 and C-5, completing 4-oxatricyclo (3.3.1.0 ^2,7^)nonane skeleton. BrasilamidesL-N (**2**–**4**) are new additions of bergamotane sesquiterpenoids, possessing the unique 9-oxatricyclo(4.3.0.0 ^4,7^)nonane skeleton. Brasilamides L (**2**) and M (**3**) are the hydrogenated and oxygenated products of brasilamide C (**6**) [13], respectively. Whereas brasilamide N (**4**) is structurally related to **6**, but differs by having a methyl group at C-1, a carboxylic carbon at C-8, and a hydroxy group at C-12 instead of an oxygenated methlene unit, a methylene unit, and a ketone carbon, respectively.

## 3. Experimental Section

### 3.1. General Experimental Procedures

Optical rotations were measured on a Perkin-Elmer 241 polarimeter, and UV data were obtained on a Shimadzu Biospec-1601 spectrophotometer. IR data were recorded using a Nicolet Magna-IR 750 spectrophotometer. ^1^H- and ^13^C-NMR data were acquired with Varian Mercury-400, -500, and -600 spectrometers using solvent signals (acetone-*d*_6_: δ_H_ 2.05/δ_C_ 29.8, 206.1) as references. The HMQC and HMBC experiments were optimized for 145.0 and 8.0 Hz, respectively. ESIMS and HRESIMS data were obtained using an Agilent Accurate-Mass-Q-TOF LC/MS 6520 instrument equipped with an electrospray ionization (ESI) source. The fragmentor and capillary voltages were kept at 125 and 3500 V, respectively. Nitrogen was supplied as the nebulizing and drying gas. The temperature of the drying gas was set at 300 °C. The flow rate of the drying gas and the pressure of the nebulizer were 10 L/min and 10 psi, respectively. All MS experiments were performed in positive ion mode. Full-scan spectra were acquired over a scan range of *m*/*z* 100−1000 at 1.03 spectra/s. HPLC separations were performed on an Agilent 1260 instrument (Agilent, Waldbronn, Germany) equipped with a variable-wavelength UV detector.

### 3.2. Fungal Material

The culture of *P. brasiliense* Verkley was isolated from branches of *Acer truncatum* Bunge on Dongling Mountain, Beijing, in March, 2005. The isolate was identified and assigned the accession number M3-3341 in L.G.’s culture collection at the Institute of Microbiology, Chinese Academy of Sciences, Beijing. The fungal strain was cultured on slants of potato dextrose agar at 25 °C for 10 days. Agar plugs were cut into small pieces (about 0.5 × 0.5 ×0.5 cm^3^) under aseptic conditions, 15 pieces were used to inoculate in three Erlenmeyer flasks (250 mL), each containing 50 mL of media (0.4% glucose, 1% malt extract, and 0.4% yeast extract), and the final pH of the media was adjusted to 6.5. After sterilization, three flasks of the inoculated media were incubated at 25 °C on a rotary shaker at 170 rpm for five days to prepare the seed culture. Spore inoculum was prepared by suspending the seed culture in sterile, distilled H_2_O to give a final spore/cell suspension of 1 × 10^6^/mL. Fermentation was carried out in 12 Fernbach flasks (500 mL), each containing 80 g of rice. Distilled H_2_O (120 mL) was added to each flask, and the contents were soaked overnight before autoclaving at 15 psi for 30 min. After cooling to room temperature, each flask was inoculated with 5.0 mL of the spore inoculum and incubated at 25 °C for 40 days.

### 3.3. Extraction and Isolation

The fermented material was extracted repeatedly with EtOAc (4 × 1.0 L), and the organic solvent was evaporated to dryness under vacuum to afford the crude extract (9.0 g), which was fractionated by silica gel VLC using petroleum ether-EtOAc gradient elution. The fraction (100 mg) eluted with 72% EtOAc was separated by Sephadex LH-20 column chromatography (CC) eluting with 1:1 CH_2_Cl_2_/MeOH. The resulting subfractions were combined and further purified by semipreparative RP HPLC (Agilent Zorbax SB-C_18_ column; 5 μm; 9.4 mm × 250 mm; 25% MeOH in H_2_O for 2 min, followed by 25%–60% over 33 min; 2 mL/min) to afford **1** (1.2 mg, *t*_R_ 25.58 min), and **4** (9.0 mg, *t*_R_ 24.82 min). Fractions (190 mg) eluted with 80% and 95% EtOAc were fractionated again by Sephadex LH-20 CC eluting with 1:1 CH_2_Cl_2_/MeOH as eluents. Purification of the resulting subfractions afforded **2** (4.0 mg, *t*_R_ 28.40 min; 30% MeOH in H_2_O for 2 min, followed by 30%–60% over 35 min; 2 mL/min), and **3** (7.0 mg, *t*_R_ 13.21 min; 25% MeOH in H_2_O for 2 min, followed by 25%–38% over 30 min; 2 mL/min).

Brasilamide K (**1**), colorless oil; ${\left[\alpha \right]}_{\text{D}}^{25}$ −9.0 (*c* 0.1, MeOH); UV (MeOH) λ_max_ (log ε) 208 (3.76) nm; IR (neat) ν_max_ 3349 (br), 2932, 1713, 1666, 1637, 1591, 1332, 1211, 1109 cm^−1^; ^1^H-, ^13^C-NMR and HMBC data see Table 1; HRESIMS *m*/*z* 280.1534 (calcd for C_15_H_22_NO_4_, 280.1543).

Brasilamide L (**2**), colorless powder; ${\left[\alpha \right]}_{\text{D}}^{25}$ +14.0 (*c* 0.1, MeOH); UV (MeOH) λ_max_ (log ε) 221 (3.70) nm; IR (neat) ν_max_ 3331 (br), 2921, 2866, 1666, 1598, 1379, 1198, 1023 cm^−1^; ^1^H-, ^13^C-NMR and HMBC data see Table 1; HRESIMS *m*/*z* 266.1743 (calcd for C_15_H_24_NO_3_, 266.1751).

Brasilamide M (**3**), colorless oil; ${\left[\alpha \right]}_{\text{D}}^{25}$ +37.0 (*c* 0.1, MeOH); UV (MeOH) λ_max_ (log *ε*) 226 (3.58) nm; IR (neat) ν_max_ 3359 (br), 2934, 2870, 1749, 1667, 1603, 1347, 1156, 1078 cm^−1^; ^1^H-, ^13^C-NMR and HMBC data see Table 2; HRESIMS *m*/*z* 294.1332 (calcd for C_15_H_20_NO_5_, 294.1336).

Brasilamide N (**4**), colorless oil; ${\left[\alpha \right]}_{\text{D}}^{25}$ +34.7 (*c* 0.5, MeOH); UV (MeOH) λ_max_ (log *ε*) 214 (3.70) nm; IR (neat) *ν*_max_ 3350 (br), 2937, 2868, 1739, 1674, 1642, 1600, 1382, 1197, 1028 cm^−1^; ^1^H-, ^13^C-NMR and HMBC data see Table 2; HRESIMS *m*/*z* 280.1546 (calcd for C_15_H_22_NO_4_, 280.1543).

### 3.4. Preparation of (R)-MTPA (**4a**) and (S)-MTPA (**4b**) Esters

A sample of **4** (1.0 mg, 0.004 mmol), (*S*)-MTPA Cl (2.0 μL, 0.011 mmol), and pyridine-*d*_5_ (0.5 mL) were allowed to react in an NMR tube at ambient temperature for 24 h. The mixture was evaporated to dryness and purified by RP HPLC (from 70% to 100% MeOH in 20 min) to afford **4a** (0.8 mg, *t*_R_ 12.01 min): colorless oil; ^1^H-NMR (acetone-*d*_6_, 500 MHz) δ 6.72 (1H, brs, NH_2_-15), 6.32 (1H, brs, NH_2_-15), 6.09 (1H, dd, *J* = 9.4, 1.5 Hz, H-13), 5.91 (1H, ddd, *J* = 9.4, 8.0, 5.5 Hz, H-12), 2.65 (1H, dd, *J* = 5.5, 5.2 Hz, H-6), 2.44 (1H, m, H-4), 2.37 (1H, m, H-5b), 2.34 (1H, m, H-2b), 2.22 (1H, m, H-11b), 2.17 (1H, m, H-3b), 2.01 (3H, d, *J* = 1.5 Hz, H_3_-16), 1.98 (1H, m, H-11a), 1.93 (1H, m, H-2a), 1.91 (1H, m, H-3a), 1.78 (1H, d, *J* = 10.3 Hz, H-5a), 1.39 (3H, s, H_3_-10); ESIMS *m*/*z* 496 [M + H]^+^.

Another sample of **4** (1.0 mg, 0.004 mmol), (*R*)-MTPA Cl (2.0 μL, 0.011 mmol), and pyridine-*d*_5_ (0.5 mL) was processed as described above for **4a** to afford **4b**, which was purified by RP HPLC (from 70% to 100% MeOH in 20 min) to afford **4b** (0.6 mg, *t*_R_ 12.61 min): colorless oil; ^1^H-NMR (acetone-*d*_6_, 500 MHz) δ 6.81 (1H, brs, NH_2_-15), 6.36 (1H, brs, NH_2_-15), 6.26 (1H, dd, *J* = 9.4, 1.5 Hz, H-13), 5.82 (1H, ddd, *J* = 9.4, 8.0, 5.5 Hz, H-12), 2.39 (1H, dd, *J* = 5.5, 5.2 Hz, H-6), 2.29 (1H, m, H-4), 2.25 (1H, m, H-5b), 2.16 (1H, m, H-2b), 2.08 (1H, m, H-11b), 1.92 (1H, m, H-3b), 2.01 (3H, d, *J* = 1.5 Hz, H_3_-16), 1.92 (1H, m, H-11a), 1.74 (1H, m, H-2a), 1.72 (1H, m, H-3a), 1.68 (1H, d, *J* = 10.3 Hz, H-5a), 1.26 (3H, s, H_3_-10); ESIMS *m*/*z* 496 [M + H]^+^.

### 3.5. MTS Assay

The MTS assay [9] was run in triplicate. In a 96-well plate, each well was plated with 2–5 × 10^3^ cells (depending on the cell multiplication rate). After cell attachment overnight, the medium was removed, and each well was treated with 100 μL of medium containing 0.1% DMSO, or appropriate concentrations of the test compounds and the positive control paclitaxel (Sigma, St. Louis, MO, USA) (100 mM as stock solution of a compound in DMSO and serial dilutions; the test compounds showed good solubility in DMSO and did not precipitate when added to the cells). The plate was incubated for 72 h at 37 °C in a humidified, 5% CO_2_ atmosphere. Proliferation assessed by adding 20 μL of MTS (Promega, Madison, WI, USA) to each well in the dark, followed by a 90 min incubation at 37 °C. The assay plate was read at 490 nm using a microplate reader.

## 4. Conclusions

This study reported brasilamides K-N (**1**–**4**), four newbergamotane sesquiterpenoids, along with previously identified sesquiterpenoids brasilamides A and C (**5** and **6**), isolated from the scale-up fermentation cultures of the plant endophytic fungus *P. brasiliense* Verkley. Moreover, the cytotoxicity of all compounds against a panel of eight human tumor cell lines were assayed. Although negative results were given, further studies still need to elucidate their promising bioactivity. The discovery of these new natural products further demonstrated that the plant endophytic fungi could be useful sources for new secondary metabolites.

## Supplementary Materials

^1^H-NMR, ^13^C-NMR, COSY, HMBC, and NOESY spectra of compounds **1**–**4** are available as supporting information. Supplementary materials can be accessed at: http://www.mdpi.com/1420-3049/20/08/14611/s1.
