# Supplementary material for: New Bergamotane Sesquiterpenoids from the Plant Endophytic Fungus Paraconiothyrium brasiliense
Source: Molecules. 2015 Aug 12;20(8):14611–20. doi: 10.3390/molecules200814611 (PMC6332149; doi:10.3390/molecules200814611)
Supplement: Supplementary file 1 [file molecules-20-14611-s001.pdf]

Supplementary Materials

Figure List

Figure S1. ^{1}H-NMR spectrum of brasilamide K (**1**; 600 MHz, acetone-d_6)

Figure S2. ^{13}C-NMR spectrum of brasilamide K (**1**; 150 MHz, acetone-d_6)

Figure S3. ^{1}H-^{1}H COSY spectrum of brasilamide K (**1**; 600 MHz, acetone-d_6)

Figure S4. HMQC spectrum of brasilamide K (**1**; 600 MHz, acetone-d_6)

Figure S5. HMBC spectrum of brasilamide K (**1**; 600 MHz, acetone-d_6)

Figure S6. NOESY spectrum of brasilamide K (**1**; 600 MHz, acetone-d_6)

Figure S7. ^{1}H-NMR spectrum of brasilamide L (**2**; 600 MHz, acetone-d_6)

Figure S8. ^{13}C-NMR spectrum of brasilamide L (**2**; 150 MHz, acetone-d_6)

Figure S9. ^{1}H-^{1}H COSY spectrum of brasilamide L (**2**; 600 MHz, acetone-d_6)

Figure S10. HMQC spectrum of brasilamide L (**2**; 600 MHz, acetone-d_6)

Figure S11. HMBC spectrum of brasilamide L (**2**; 600 MHz, acetone-d_6)

Figure S12. NOESY spectrum of brasilamide L (**2**; 600 MHz, acetone-d_6)

Figure S13. ^{1}H-NMR spectrum of brasilamide M (**3**; 400 MHz, acetone-d_6)

Figure S14. ^{13}C-NMR spectrum of brasilamide M (**3**; 100 MHz, acetone-d_6)

Figure S15. ^{1}H-^{1}H COSY spectrum of brasilamide M (**3**; 400 MHz, acetone-d_6)

Figure S16. HMQC spectrum of brasilamide M (**3**; 400 MHz, acetone-d_6)

Figure S17. HMBC spectrum of brasilamide M (**3**; 400 MHz, acetone-d_6)

Figure S18. NOESY spectrum of brasilamide M (**3**; 400 MHz, acetone-d_6)

Figure S19. ^{1}H-NMR spectrum of brasilamide N (**4**; 500 MHz, acetone-d_6)

Figure S20. ^{13}C-NMR spectrum of brasilamide N (**4**; 125 MHz, acetone-d_6)

Figure S21. ^{1}H-^{1}H COSY spectrum of brasilamide N (**4**; 500 MHz, acetone-d_6)

Figure S22. HMQC spectrum of brasilamide N (**4**; 500 MHz, acetone-d_6)

Figure S23. HMBC spectrum of brasilamide N (**4**; 500 MHz, acetone-d_6)

Figure S24. NOESY spectrum of brasilamide N (**4**; 500 MHz, acetone-d_6)

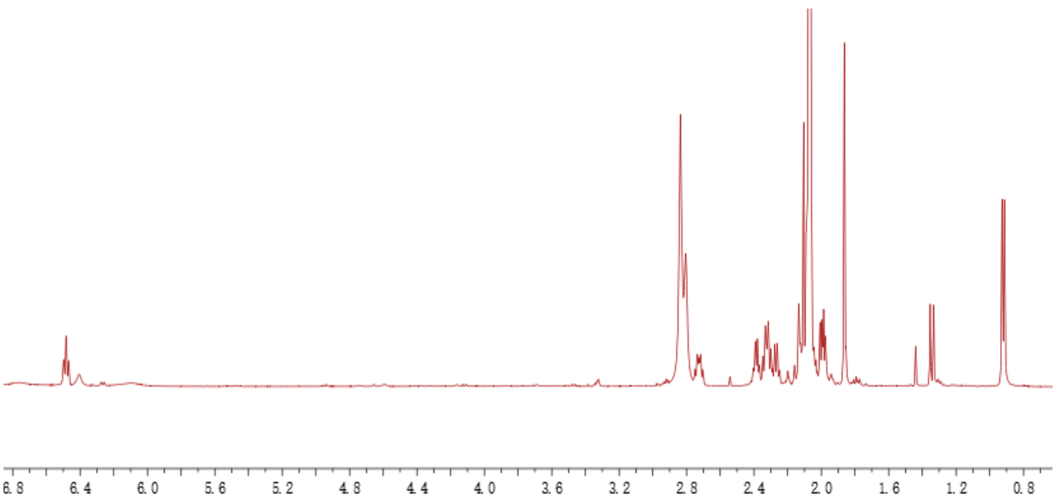

Figure S1. ^{1}H-NMR spectrum of brasilamide K (**1**; 600 MHz, acetone-d_6).

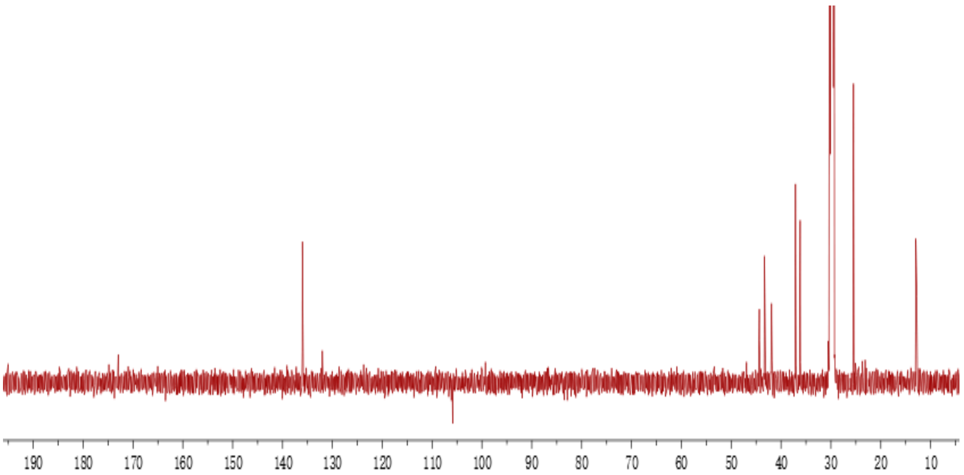

Figure S2. ^{13}C-NMR spectrum of brasilamide K (**1**; 150 MHz, acetone-d_6).

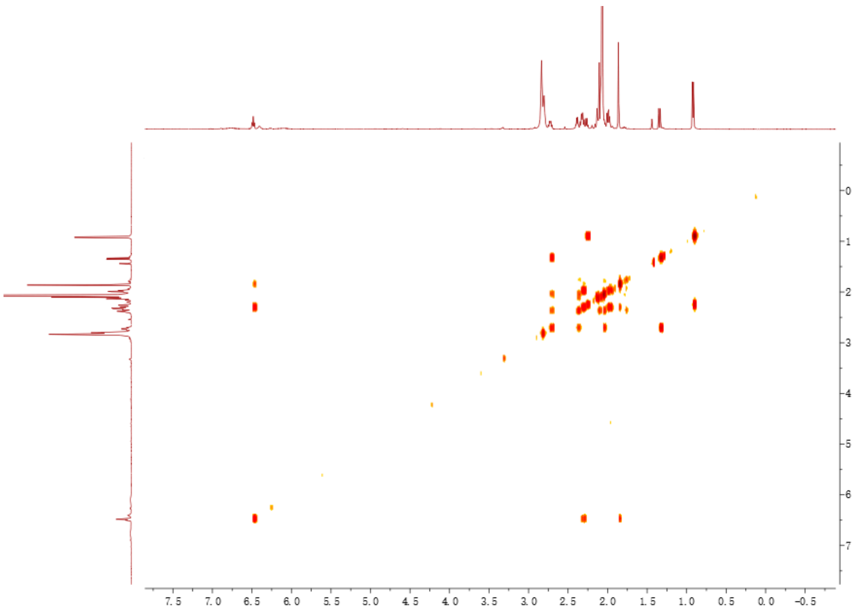

Figure S3. ^{1}H-^{1}H COSY spectrum of brasilamide K (**1**; 600 MHz, acetone-d_6).

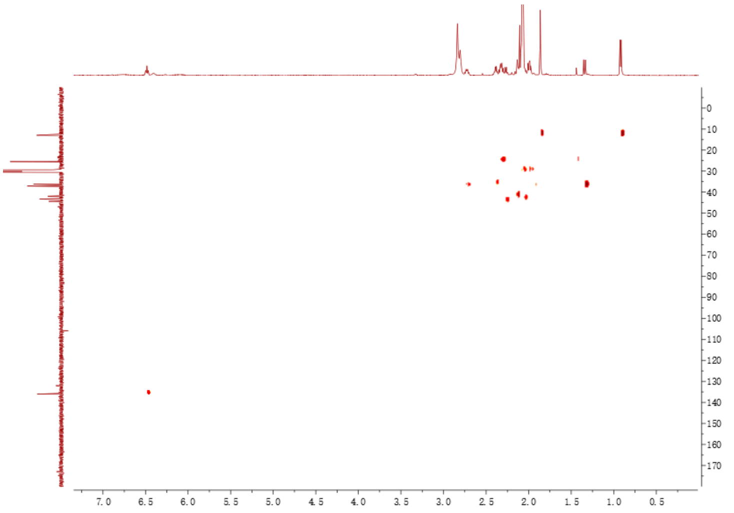

Figure S4. HMQC spectrum of brasilamide K (**1**; 600 MHz, acetone-d_6).

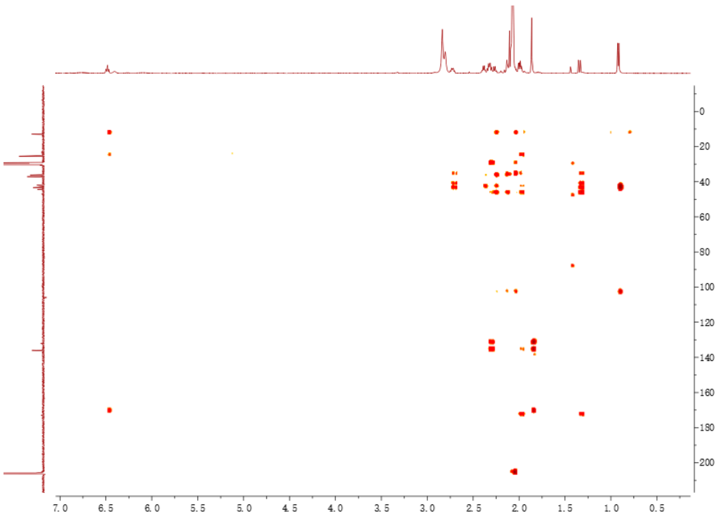

Figure S5. HMBC spectrum of brasilamide K (**1**; 600 MHz, acetone-d_6).

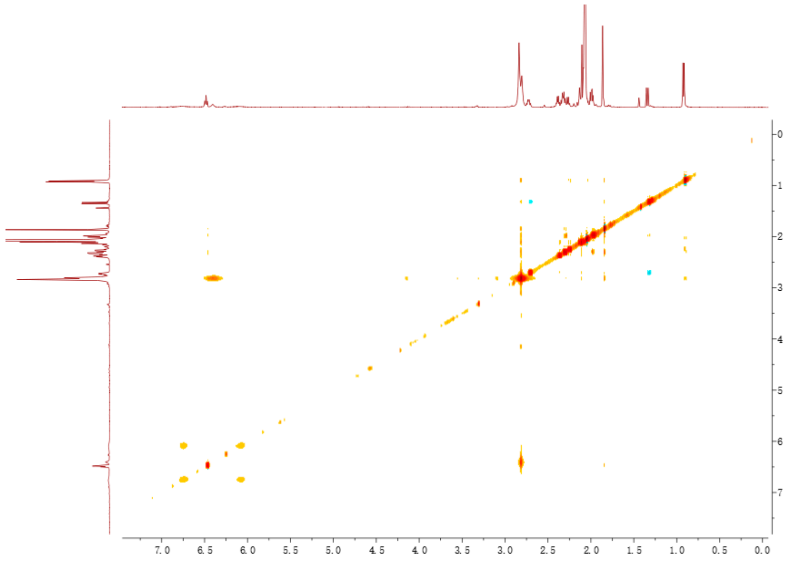

Figure S6. NOESY spectrum of brasilamide K (**1**; 600 MHz, acetone-d_6).

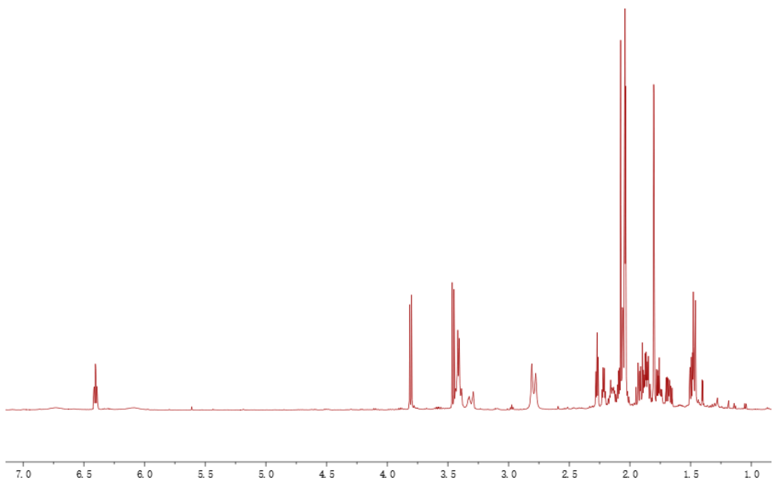

Figure S7. ^{1}H-NMR spectrum of brasilamide L (**2**; 600 MHz, acetone-d_6).

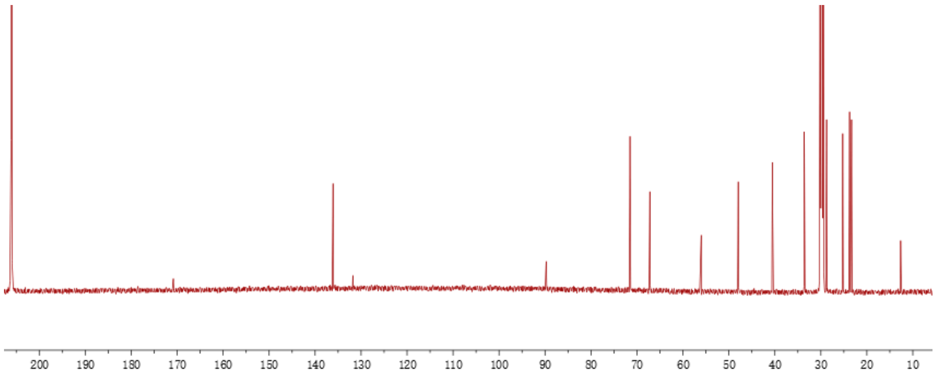

Figure S8. ^{13}C-NMR spectrum of brasilamide L (**2**; 150 MHz, acetone-d_6).

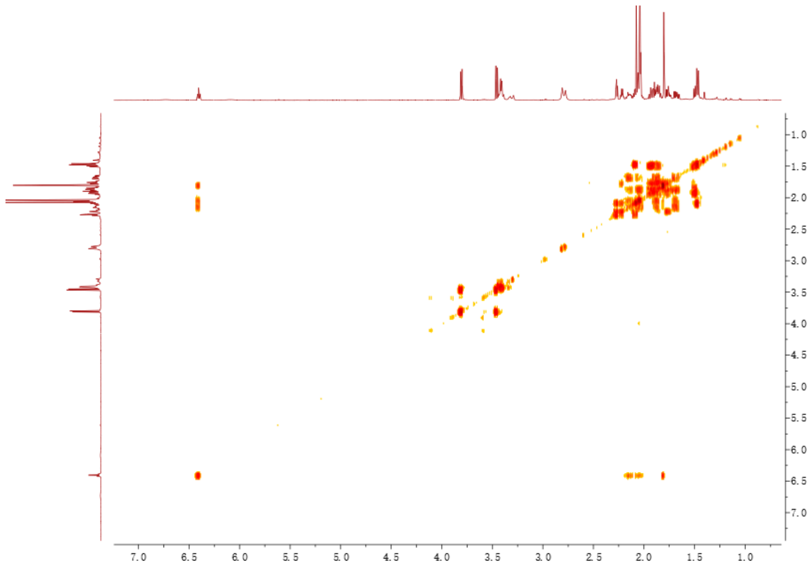

Figure S9. ^{1}H-^{1}H COSY spectrum of brasilamide L (**2**; 600 MHz, acetone-d_6).

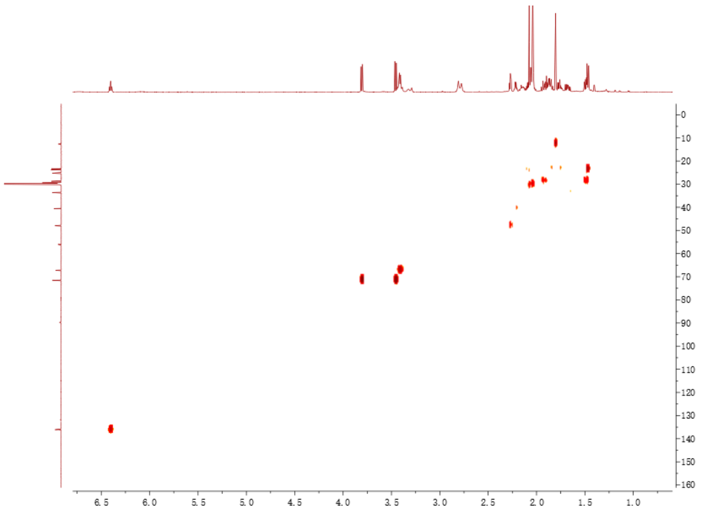

Figure S10. HMQC spectrum of brasilamide L (**2**; 600 MHz, acetone-*d*6).

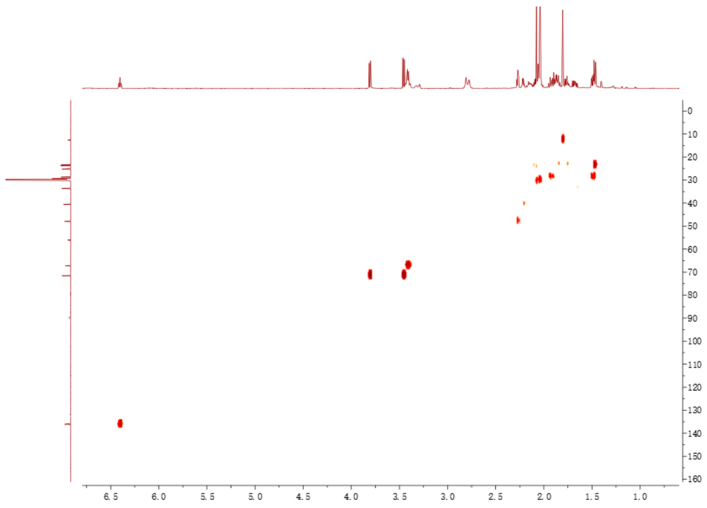

Figure S11. HMBC spectrum of brasilamide L (**2**; 600 MHz, acetone-*d*6).

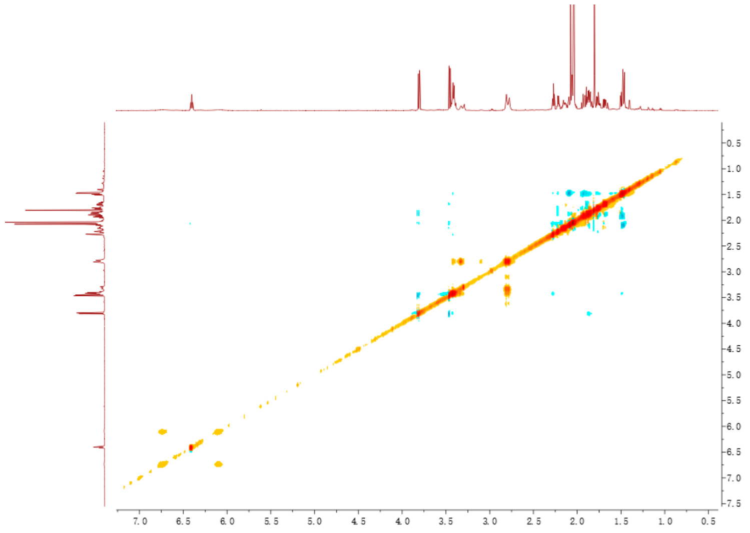

Figure S12. NOESY spectrum of brasilamide L (**2**; 600 MHz, acetone-*d*6).

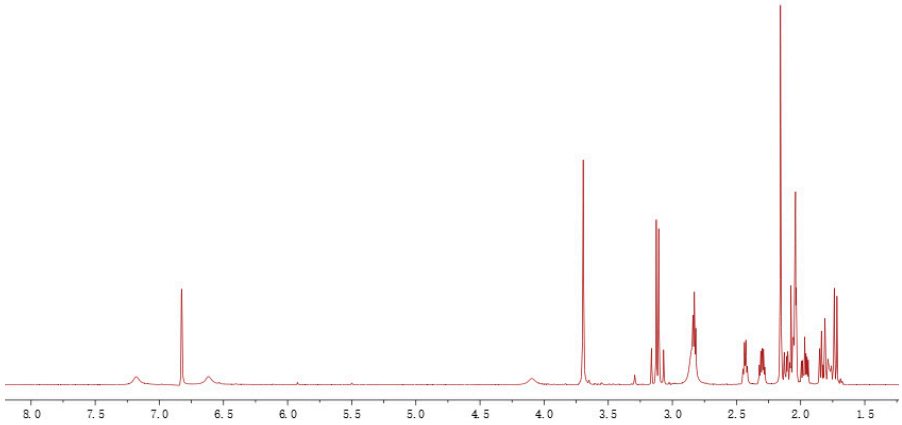

Figure S13. ^{1}H-NMR spectrum of brasilamide M (**3**; 400 MHz, acetone-d_6).

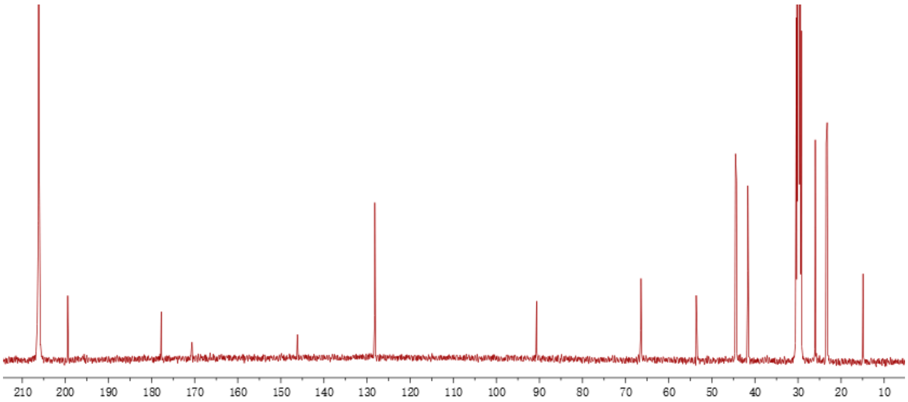

Figure S14. ^{13}C-NMR spectrum of brasilamide M (**3**; 100 MHz, acetone-d_6).

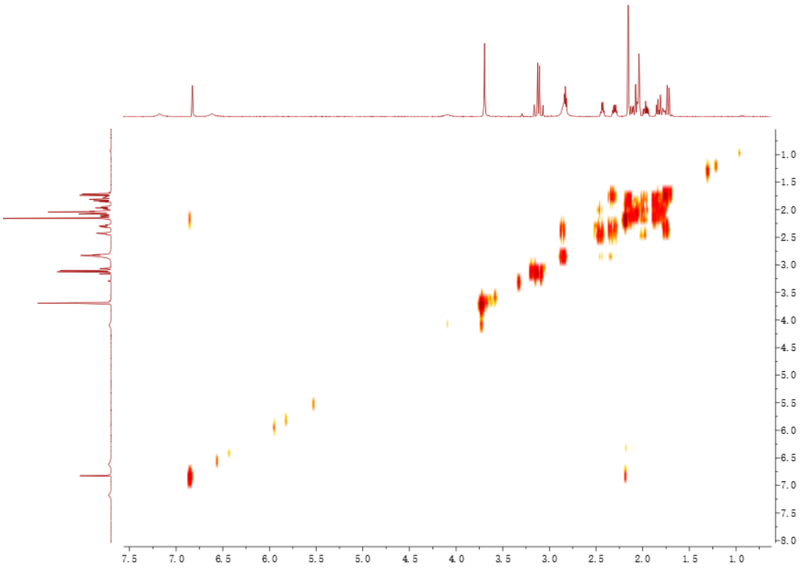

Figure S15. ^{1}H-^{1}H COSY spectrum of brasilamide M (**3**; 400 MHz, acetone-d_6).

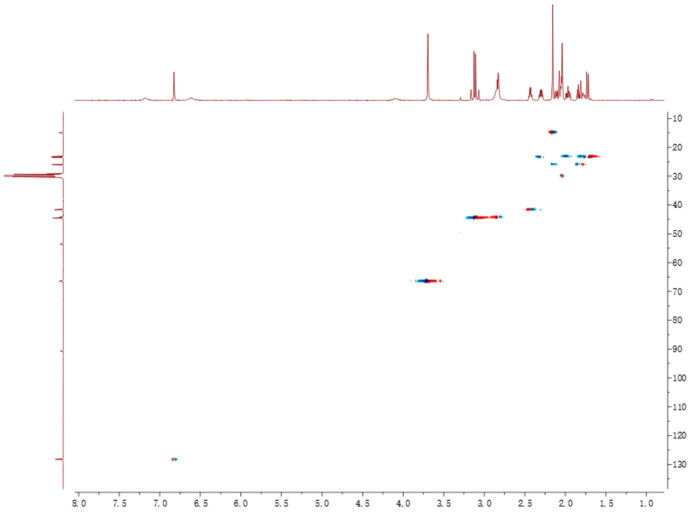

Figure S16. HMQC spectrum of brasilamide M (**3**; 400 MHz, acetone-d_6).

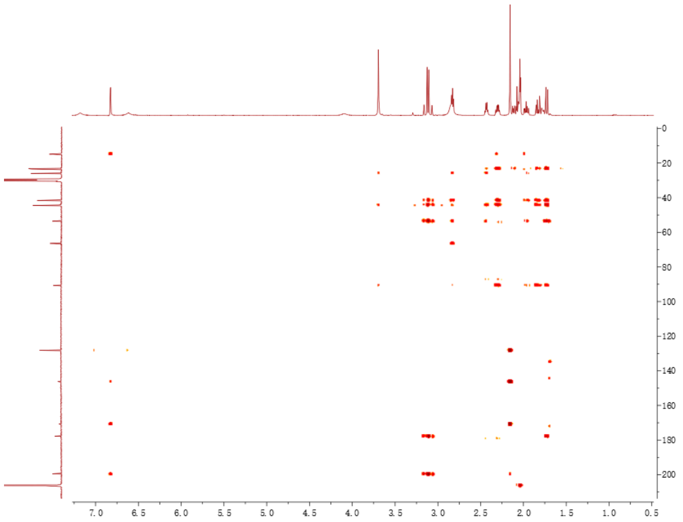

Figure S17. HMBC spectrum of brasilamide M (**3**; 400 MHz, acetone-d_6).

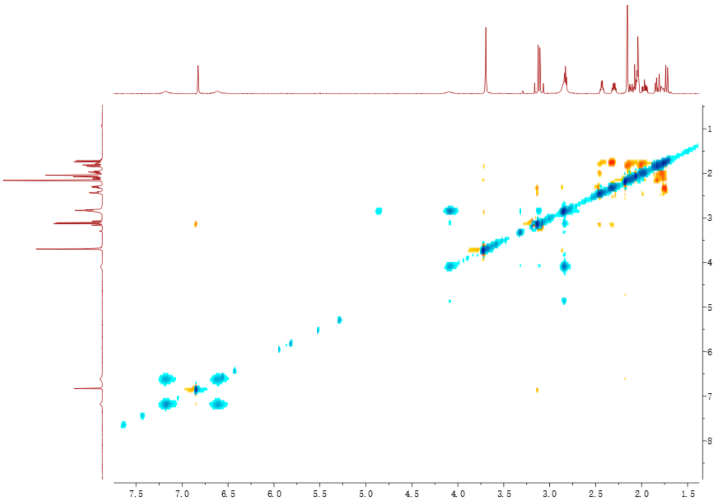

Figure S18. NOESY spectrum of brasilamide M (**3**; 400 MHz, acetone-d_6).

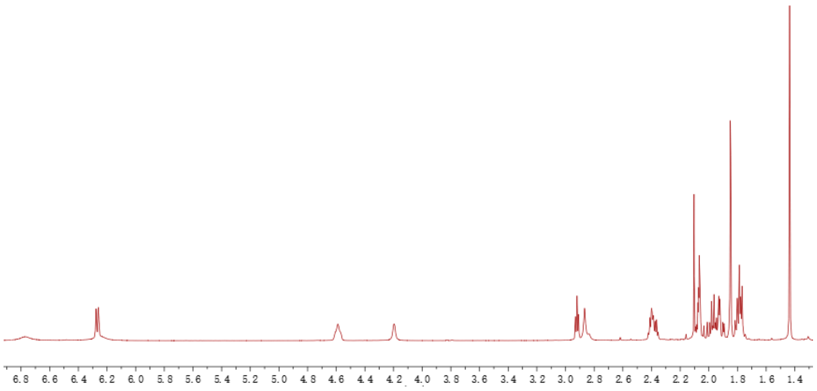

Figure S19. ^{1}H-NMR spectrum of brasilamide N (**4**; 500 MHz, acetone-d_6).

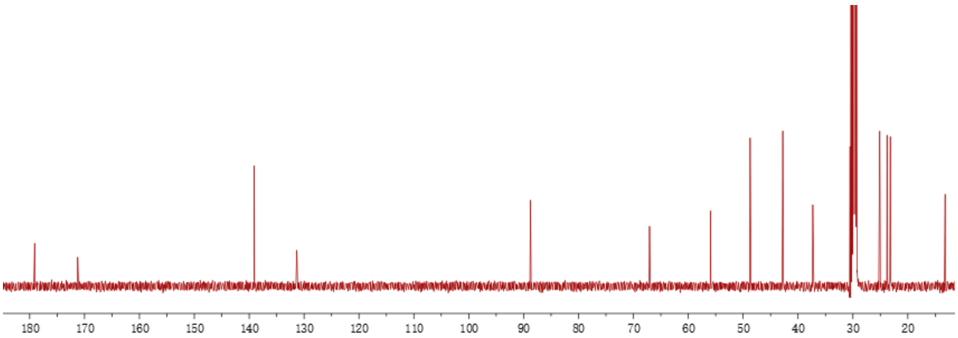

Figure S20. ^{13}C-NMR spectrum of brasilamide N (**4**; 125 MHz, acetone-d_6).

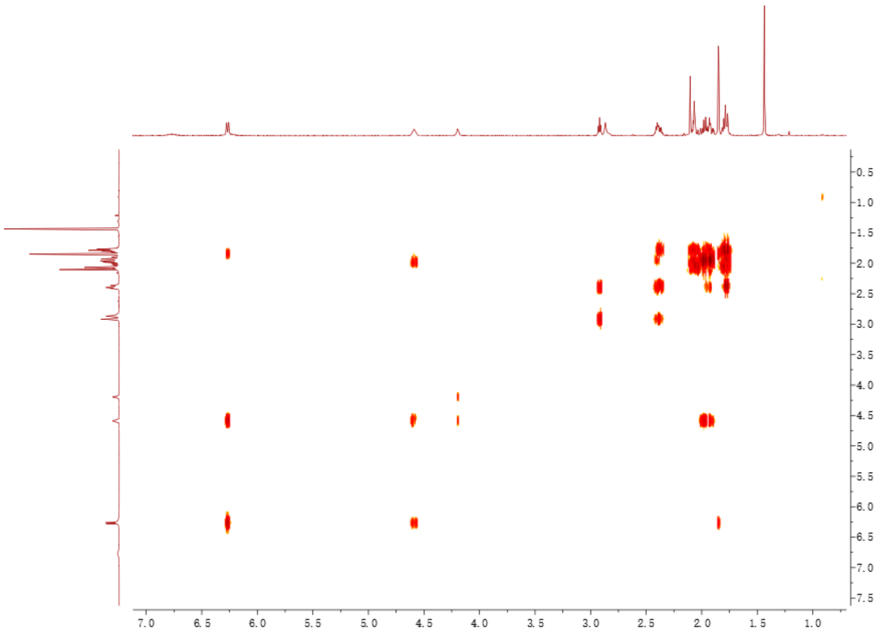

Figure S21. ^{1}H-^{1}H COSY spectrum of brasilamide N (**4**; 500 MHz, acetone-d_6).

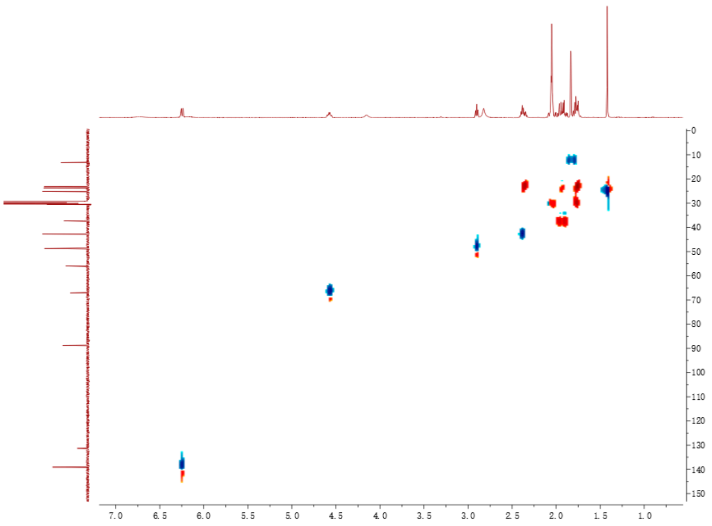

Figure S22. HMQC spectrum of brasilamide N (**4**; 500 MHz, acetone-d_6).

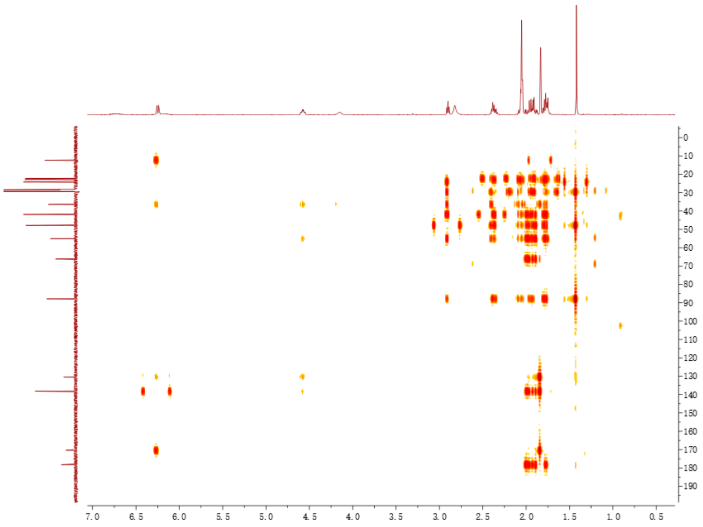

Figure S23. HMBC spectrum of brasilamide N (**4**; 500 MHz, acetone-d_6).

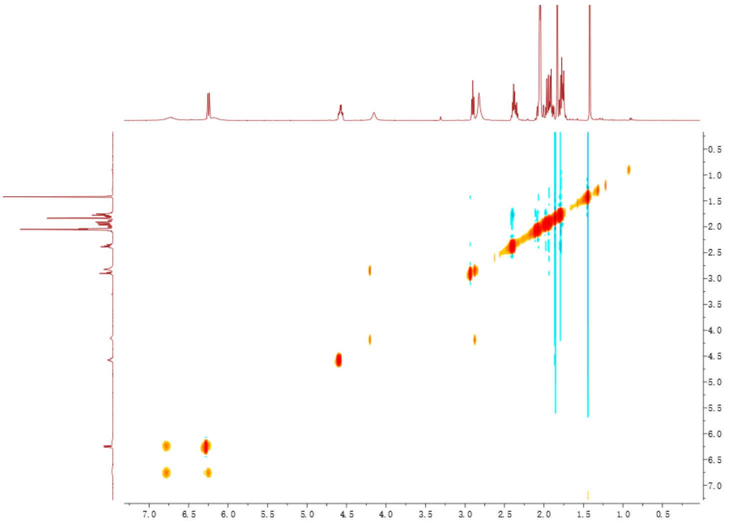

Figure S24. NOESY spectrum of brasilamide N (**4**; 500 MHz, acetone-d_6).
